# Investigation of A Slow-Light Enhanced Near-Infrared Absorption Spectroscopic Gas Sensor, Based on Hollow-Core Photonic Band-Gap Fiber

**DOI:** 10.3390/s18072192

**Published:** 2018-07-07

**Authors:** Zhi-Fa Wu, Chuan-Tao Zheng, Zhi-Wei Liu, Dan Yao, Wen-Xue Zheng, Yi-Ding Wang, Fei Wang, Da-Ming Zhang

**Affiliations:** State Key Laboratory of Integrated Optoelectronics, College of Electronic Science and Engineering, 2699 Qianjin Street, Jilin University, Changchun 130012, China; wuzf@jlu.edu.cn (Z.-F.W); liuzw16@mails.jlu.edu.cn (Z.-W.L.); yaodan17@mails.jlu.edu.cn (D.Y.); wenxue.zheng@foxmail.com (W.-X.Z.); ydwang@jlu.edu.cn (Y.-D.W.); zhangdm@jlu.edu.cn (D.-M.Z.)

**Keywords:** photonic band-gap fiber, infrared absorption spectroscopy, gas sensor, slow light

## Abstract

Generic modeling and analysis of a slow-light enhanced absorption spectroscopic gas sensor was proposed, using a mode-tuned, hollow-core, photonic band-gap fiber (HC-PBF) as an absorption gas cell. Mode characteristics of the un-infiltrated and infiltrated HC-PBF and gas absorption enhancement of the infiltrated HC-PBF were analyzed. A general rule of microfluidic parameters for targeting different gas species in the near-infrared was obtained. Ammonia (NH_3_) was used as an example to explore the effects of slow light on gas detection. The second harmonic (2*f*) signal and Allan deviation were theoretically investigated based on the derived formulations.

## 1. Introduction

The sensitive and selective detection of trace chemicals is important for environmental, safety, and industrial process monitoring, as well as in national security applications. Infrared absorption spectroscopy, which relies on the absorption lines of a gas molecule for identifying and detecting trace chemicals, is suitable for gas detection [1,2]. Conventional absorption spectroscopic sensor systems [3,4,5], such as cavity-enhanced absorption spectroscopy, are ultrasensitive, but are large and have a heavy tabletop instrument that requires a large gas cell with a long absorption path length. Recently, there has been an increasing emphasis on the miniaturization of chemical analysis systems and optics, such as lab-on-a-chip microsystems [6,7,8] and fiber optic sensors [9,10]. In particular, miniaturized gas sensors based on photonic hollow-core crystal (PHC) waveguides are receiving attention for achieving superior performance in optical signal processing and sensing applications [11,12,13]. Intuitively, slow-light propagation offers photons more time and increases the effective optical length for interacting with a host medium (e.g., gas molecules) [14,15].

Infrared absorption spectroscopy is based on the absorption of a gas molecule on an infrared light with a specific wavelength. Different molecules have different absorption wavelengths, leading to the unique selectivity of the technique. Therefore, this technique is particularly suitable for the detection of gas mixtures. Silicon PHC slot waveguide gas sensors based on the Beer-Lambert law have been demonstrated [7,11]. Besides, a hollow-core photonic band-gap fiber (HC-PBF) allows the confinement of an optical mode and chemical materials simultaneously within the hollow core and provides an ideal platform for strong light-matter interactions over a long distance [15]. Thus, there are a wide variety of gas sensors based on HC-PBFs for the detection of different gas species or for the application of different signal processing techniques. PHC or HC-PBF sensors have unique dispersive properties that allow for the control and manipulation of light-matter interactions on the length scale of the light wavelength, by infiltrating optical fluids into selected air holes of PHC devices, which makes it possible to tune and reconfigure photonic devices suitable for various applications.

In view of the above considerations, as the main focus and novelty of the paper, we propose a generic design and analysis of a slow-light enhanced absorption spectroscopic gas sensor based on HC-PBF. The idea and simulation of selective infiltration of HC-PBF to tune the wavelength of the maximum enhancement factor for specific modes are proposed. The microfluidic parameters for targeting different gas species are obtained. Ammonia (NH_3_) is used as an example to explore the effects of slow light on gas detection as a reference in the design and development of such kind of gas sensors.

## 2. Modeling of the Hollow-Core Photonic Band-Gap Fiber-Based Spectroscopic Gas Sensor

**Figure 1:**
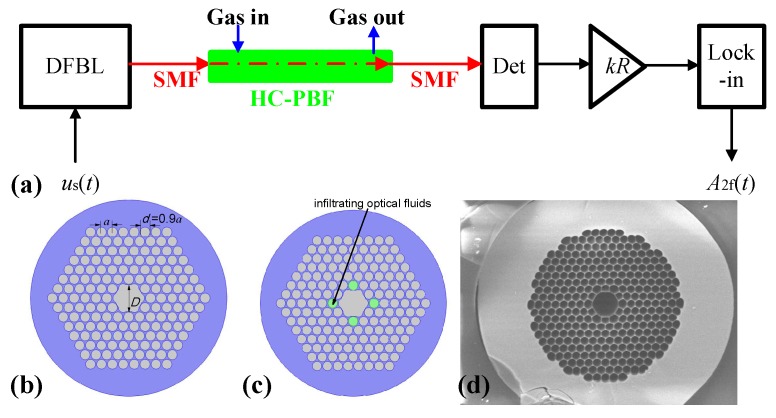
(**a**) Schematic of a hollow-core photonic band-gap fiber (HC-PBF)-based spectroscopic gas sensor. The sub-figures show (**b**) the cross-section structure by un-infiltrating optical fluids to the HC-PBF, and (**c**) the cross-section structure by infiltrating optical fluids into the selected air holes of the HC-PBF. (**d**) Scanning electron microscope (SEM) image of the HC-PBF commercially available from NKT optics.

A simplified schematic of an HC-PBF-based spectroscopic gas sensor is shown in Figure 1a, which includes a tunable distributed feedback laser (DFBL), two pieces of single-mode fiber (SMF), a HC-PBF for gas sensing, an infrared detector (Det), a pre-amplifier (*kR*), and a lock-in amplifier (Lock-in). Figure 1b,c show the two-dimensional (2-D) cross-section view of an HC-PBF with a lattice constant *a*, an air hole diameter of d=0.9a, and a hollow core diameter of D=2.52a. The lattice of air holes in the simulation is definite, which is similar to the structure shown in the scanning electron microscope (SEM) image of the fiber in Figure 1d, commercially available from NKT Photonics (Birkerød, Denmark). The gray circles represent the hollow air holes and core, while the blue regions represent the chalcogenide glass (ChG) with a linear refractive index of *n_0_* = 2.8.

The principle of infrared absorption spectroscopy is based on the Beer-Lambert law [4,5], given by:

(1) $$I\left(\lambda \right)=I_{0}\left(\lambda \right)\exp\left[-\gamma \left(\lambda \right)\alpha \left(\lambda \right)CL\right],$$

where *I*_0_ is the incident light intensity; α(*λ*) is the gas absorption coefficient at the wavelength of *λ*; *C* is the gas concentration; *L* is the optical interaction length; and *γ* is a wavelength-dependent, medium-specific absorption enhancement factor, due to slow-light effect in dispersive structures. In conventional free-space systems, *γ* = 1. For PHC devices, the enhancement factor *γ* is

(2) $$\gamma =f\times \frac{c/n}{v_{g}},$$

where *f* is a filling factor denoting the relative fraction of the optical field, *c* is the light wave velocity in free space, and $v_{g}=\frac{d\omega }{d\beta }=c/n_{g}$ is the light wave group velocity. From Equation (2), the enhancement factor can obviously be increased by either a small $v_{g}$ or a high *f*.

Due to the use of high-frequency wavelength modulation for 1/f noise suppression, and a lock-in amplifier for further noise suppression, wavelength modulation spectroscopy (WMS) is more sensitive than the direct absorption spectroscopy (DAS) technique. Therefore, the WMS technique is usually adopted in an infrared gas sensor system. In this work, WMS is used as an example, to show how this enhancement will affect the second harmonic signal. This technique requires that the emitting wavelength of the laser must be stabilized, in order to be the peak absorption wavelength for a certain gas molecule.

A periodic saw-tooth signal is used to scan the absorption line, which can be expressed in one period as:

(3) $$u_{\mathrm{saw}}(t)=A_{\mathrm{saw}}+\frac{A_{\mathrm{saw}}}{T_{\mathrm{saw}}}(t-T_{\mathrm{saw}}),\;0\leq t\leq T_{\mathrm{saw}},$$

where *A*_saw_ and *T*_saw_ are the amplitude and period of the sawtooth signal, respectively. Also, a sinewave signal, expressed as:

(4) $$u_{\sin}(t)=\frac{A_{\sin}}{2}\sin(\omega_{\sin}t),$$

is used to modulate the laser, where *A*_sin_ and *ω*_sin_ are the amplitude and the angular frequency of the sinewave signal, respectively. Thus, the total driving signal of the DFBL is

(5) $$u_{s}(t)=u_{\mathrm{saw}}(t)+u_{\sin}(t).$$

Under the operation of *u*(*t*), the variation of the emitting wavelength leads to a change of the absorption coefficient *α*(*t*), expressed as [5]:

(6) $$\alpha (t)=\frac{\alpha_{0}}{1+{\left[\left(\upsilon_{0}+\delta u(t)-\upsilon_{g}\right)/\gamma^{\prime }\right]}^{2}},$$

where *υ*_0_ is the central emitting wavenumber (determined by the laser operating temperature), *υ*_g_ is the central absorption wavenumber of the targeted gas molecule, *α*_0_ is the gas absorption coefficient at *υ*_0_, *γ*′ is the half-width of the absorption peak at *υ*_0_, and *δ* is a light modulation coefficient. The laser power can be expressed as Equation (7):

(7) $$P_{t}(t)=K_{\mathrm{EO}}Gu(t),$$

where *G* is a voltage-to-current conversion coefficient of the laser driver, and *K*_EO_ is a voltage-to-optical conversion coefficient of the laser. The SMF will couple with the HC-PBF for gas sensing. Let the normalized mode field distribution of the SMF be E_SMF_. The mode coupling coefficient *η*_m_ between SMF and the *m*-th guided mode of the HC-PBF can be determined by Equation (8):

(8) $$\eta_{m}=\frac{\iint E_{\mathrm{SMF}}^{*}E_{\mathrm{PBF}}^{m}dxdy}{\iint {\left|E_{\mathrm{PBF}}^{m}\right|}^{2}dxdy}.$$

Then, with two coupling effects between the SMF and the HC-PBF, the input power to the second SMF without gas absorption is given by Equation (9):

(9) $$P_{t,\mathrm{PBF}}(t)=P_{t}(t)\sum_{i}\eta_{m}^{2}.$$

Considering a slow-light induced gas absorption loss, the input power to the second SMF is

(10) $$P_{r}\left(t\right)=P_{t}\left(t\right)\sum_{i}\eta_{m}^{2}\exp\left[-\gamma_{m}\left(t\right)\alpha \left(t\right)CL\right],$$

where $\gamma_{m}\left(t\right)$ is a time-dependent enhancement factor of the *m*-th HC-PBF mode. The detector signal from the pre-amplifier can be expressed as:

(11) $$u_{d}(t)=kRK_{\mathrm{OE}}K_{\mathrm{EO}}Gu_{s}(t)\sum_{i}\eta_{m}^{2}\exp\left[-\gamma_{m}\left(t\right)\alpha \left(t\right)CL\right],$$

where *kR* is the gain of the pre-amplifier, and $K_{\mathrm{OE}}$ is the optical-to-electrical conversion coefficient of the infrared detector. Next, we use an orthogonal lock-in amplifier to extract the 2*f* signal from $u_{d}(t)$, as:

(12) $$A_{2f}\left(t\right)=\sqrt{{\left(A_{2f,⊥}\right)}^{2}+{\left(A_{2f,∥}\right)}^{2}},$$

where $A_{2f,⊥}\left(t\right)=\int_{t-T_{\mathrm{int}2}}^{t}u_{d}(\tau )\sin\left(2\omega_{\sin}\tau \right)d\tau$, $A_{2f,∥}\left(t\right)=\int_{t-T_{\mathrm{int}2}}^{t}u_{d}(\tau )\cos\left(2\omega_{\sin}\tau \right)d\tau$ and $T_{\mathrm{int}2}$ is an integral time factor, determined by the cutoff frequency of the related low-pass filter.

## 3. Mode Analysis of Hollow-Core Photonic Band-Gap Fiber

### 3.1. Uninfiltrated Hollow-Core Photonic Band-Gap Fiber Mode Analysis

**Figure 2:**
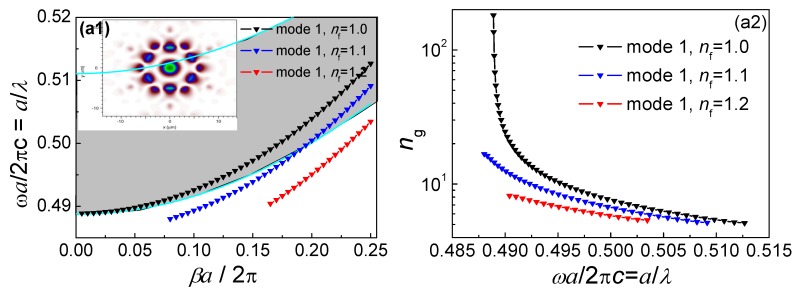

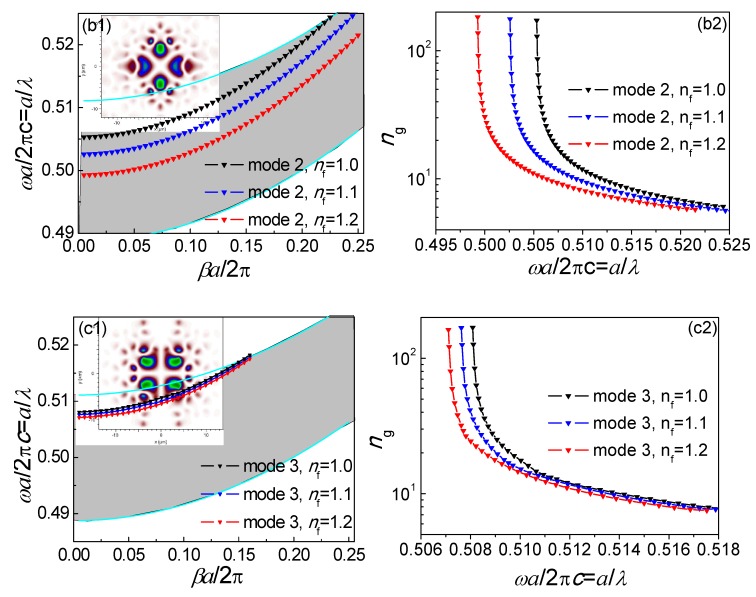
Photonic band gap and group index of infiltrated HC-PBF; (**a1**,**a2**) mode 1, (**b1**,**b2**) mode 2, and (**c1**,**c2**) mode 3. Field distributions of guided optical modes 1–3 in the HC-PBF with slow-light effect are also shown in Figures a1, b1, and c1, respectively.

**Figure 3:**
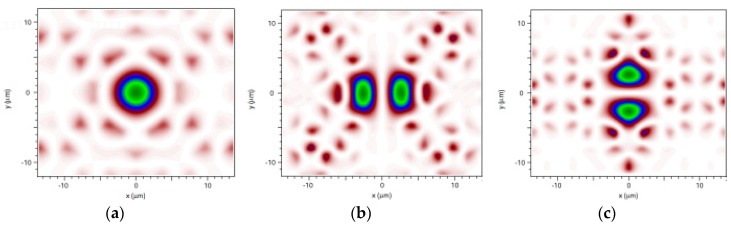
Field distributions of guided optical modes 4–6 in the HC-PBF without slow-light effect are shown in Figures (**a**), (**b**), and (**c**), respectively.

Based on the commercial software Comsol 5.0, by using the finite element method (FEM)-based plane wave expansion code to solve the Maxwell’s equations, it is feasible to obtain the photonic band structure for the un-infiltrated PBF. The gray region extending over the normalized frequency range of 0.4877 ≤ *a*/*λ* ≤ 0.51105 represents the photonic band gap (PBG) region of the HC-PBF. Six modes were found to be confined along the fiber hollow core, as shown in Figure 2(a1,b1,c1), as well as Figure 3a–c. Among these modes, modes 4, 5, and 6 (Figure 3a–c) cannot be confined when propagation constant *β*→0, indicating that they do not possess a slow-light property. As seen in Figure 2(a1,b1,c1), the three black curves, indicated by triangles in the PBG area, represent the dispersion relation of the three guided optical modes for the zero propagation constant (*βα*/2*π* = 0). Figure 2(a2,b2,c2) show the group index *n*_g_ of the three modes, which will reach a peak propagation constant *β*→0.

### 3.2. Infiltrated Hollow-Core Photonic Band-Gap Fiber Mode Analysis

Optical fluids with different indices (*n*_f_ = 1.0, 1.1, 1.2) were infiltrated into the selected green air holes, which can be realized by using the selective infiltration methods presented in [16,17]. The dispersion relations and the group index of modes 1, 2, and 3 for infiltrating fluids with different indices were calculated, and are shown in Figure 2a–c. The slow-light region shifted to a long wavelength by increasing the refractive index of optical fluids. At the band edge, the group indices increased sharply; correspondingly, the group velocity was far less than the speed of light in free space, and a large group velocity dispersion (GVD) occurred. Mode 1 got completely out of the PBG region with infiltrating fluids, and thus only modes 2 and 3 are considered. For a given fluid, however, the wavelength shift experienced by mode 2 was significantly larger than that of mode 3. This results from the fact that the electrical field intensity of mode 2 in the selected air holes with infiltrated fluids was stronger than that of mode 3. Different degrees of shift led to different degrees of wavelength tuning: mode 2 is suitable for coarse tuning, and mode 3 is suitable for fine tuning of a slow-light region. Without the need to change the structural parameters of the fiber, the guided modes of an appropriately designed slow-light regime by use of selectively infiltrated HC-PBF can be tuned to the absorption line of a target gas species as desired for maximizing the gas absorption.

### 3.3. Slow-Light Effect Analysis

The HC-PBF is filled with a homogeneously distributed gas with a complex refractive index of $n=n^{\prime }+n^{\prime \prime }=1+n^{\prime \prime }\left(n^{\prime }=1\right)$, where the imaginary part *n*″ is an extinction coefficient that relates to fiber absorption loss. The relationship between the extinction coefficient $n^{\prime \prime }$ and the gas absorption loss coefficient $\alpha_{g}\left(\lambda \right)$ is as follows:

(13) $$\alpha_{g}\left(\lambda \right)=2kn^{\prime \prime }=\frac{4\pi }{\lambda }n^{\prime \prime }.$$

**Figure 4:**
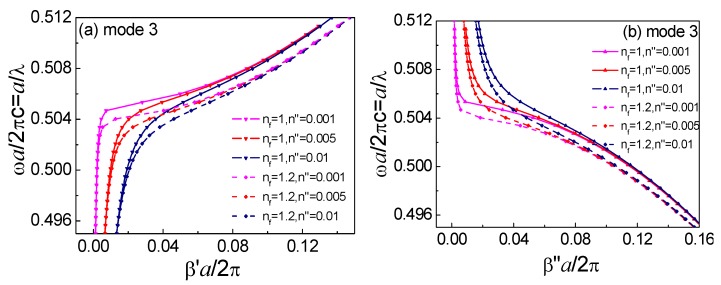
Complex propagation constants versus the normalized frequency α/λ for mode 3 of the un-infiltrated and infiltrated HC-PBF of Figure 1, (**a**) β′, and (**b**) β″. Optical fluids ($n_{f}=1.0,\;1.2$) are infiltrated into the selected air holes (Figure 1). Other un-infiltrated air holes and the core are filled with gas, with a complex refractive index of $n=n^{\prime }+n^{\prime \prime }=1+n^{\prime \prime }$ and $n^{\prime \prime }$ = 0.001, 0.005, 0.01.

By using finite element method (FEM) to numerically solve the wave equation:

(14) $$\nabla \times \left(\nabla \times E\right)-K_{0}^{2}\varepsilon_{\gamma }E=0,\;\varepsilon_{\gamma }={\left(n^{\prime }+in^{\prime \prime }\right)}^{2},$$

we can calculate the complex propagation constants $\beta =\beta^{\prime }+i\beta^{\prime \prime }$ and obtain the absorption loss of an infiltrated HC-PBF. The relationships between the complex propagation constants $\beta =\beta^{\prime }+i\beta^{\prime \prime }$ and the normalized frequency α/λ for mode 3 of the un-infiltrated and infiltrated HC-PBF are shown in Figure 4. From Figure 4a, the dispersion departed significantly from the ideal lossless case in the assumed slow-light regime near $\beta^{\prime }\to 0$. The dispersion relation curve dropped sharply when $\beta^{\prime }\to 0$, even for a lossless, un-infiltrated HC-PBF. Figure 4b shows that the HC-PBF with fluid infiltration exhibited a lower optical loss, resulting from a smaller light-gas interaction area (except for the green air holes). With an increase of the extinction coefficient, the degrees of curvature of $\beta^{\prime }$ and $\beta^{\prime \prime }$ became flatter, and the two curves became the same value for the same $n_{f}$, with an increasingly complex propagation constant *β*. Furthermore, for the infiltrated structure, both $\beta^{\prime }$ and $\beta^{\prime \prime }$ curves shifted to a lower frequency. Through theoretical calculation, the influences of infiltrated liquid loss can be neglected, due to a very small overlap area of the four liquid holes with the six guided modes in the HC-PBF, which enables such a structure to be suitable for practical operation. 

Our focus is to explore to what extent a large group index will enhance light-matter interactions, and to investigate the absorbance enhancement factor *γ* in Equation (2). The group index *n*_g_ can be derived from the real part *β*′, and the filling factor *f* can be calculated from the modal optical electrical field distributions. However, *γ* can also be calculated based on Equation (15), as

(15) $$\gamma =\frac{\beta^{\prime \prime }-\beta^{\prime \prime }\left(n^{\prime \prime }=0\right)}{n^{\prime \prime }}$$

**Figure 5:**
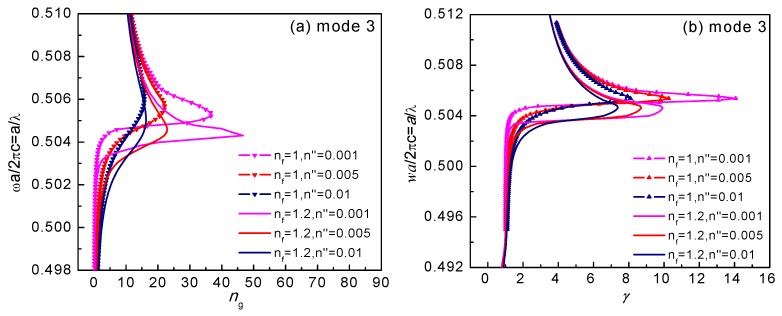
For mode 3, curves of (**a**) group index $n_{g}$, and (**b**) absorption enhancement factor γ versus the normalized frequency.

Figure 5 shows the corresponding absorption enhancement factor *γ* along with the group index *n*_g_. The variation of *γ* is similar to *n*_g_. There is a saturated value for *γ* or *n*_g_, compared to the curve of *n*_g_ in Figure 2 for a lossless structure. Under certain conditions for $n^{\prime \prime }$, there is a maximum *γ* at a specific wavelength. The weaker the intrinsic absorption of the gas is, the larger the absorption enhancement factor that can be achieved. Hence, HC-PBF with a slow-light effect exhibits a superior performance for the detection of weakly absorbing trace gases. For mode 2, similar results can be obtained.

## 4. Slow-Light Enhanced Gas Sensing Performance Analysis

### 4.1. Mode Tuning for Near-Infrared Gas Sensing

**Figure 6:**
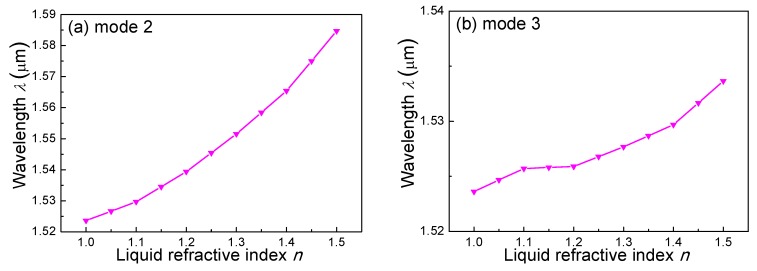
The relationship between the enhanced absorption wavelength and the refractive index of the infiltrated liquid for (**a**) mode 2 and (**b**) mode 3.

For practical applications, it is important to tune the slow-light region of a fiber mode to a specific gas absorption line. A general rule should be applied, in order to obtain an enhanced absorption wavelength (i.e., the wavelength corresponding to the peak *n*_g_ or *γ*). Figure 6a,b shows the relationship between the enhanced absorption wavelength and the refractive index of the liquid for modes 2 and 3, respectively. With an increasing liquid index, the enhanced wavelength increased from 1.51 to 1.59 µm for mode 2, so that several gas species could be detected, e.g., CO at 1.56 µm, C_2_H_2_ and NH_3_ at 1.53 µm, and CO_2_ at 1.57 µm. However, for mode 3, the enhanced wavelength only increased over a small range, i.e., from 1.523 to 1.535 µm, which is suitable for a fine wavelength tuning.

### 4.2. NH_3_ Absorption Enhancement Analysis

**Figure 7:**
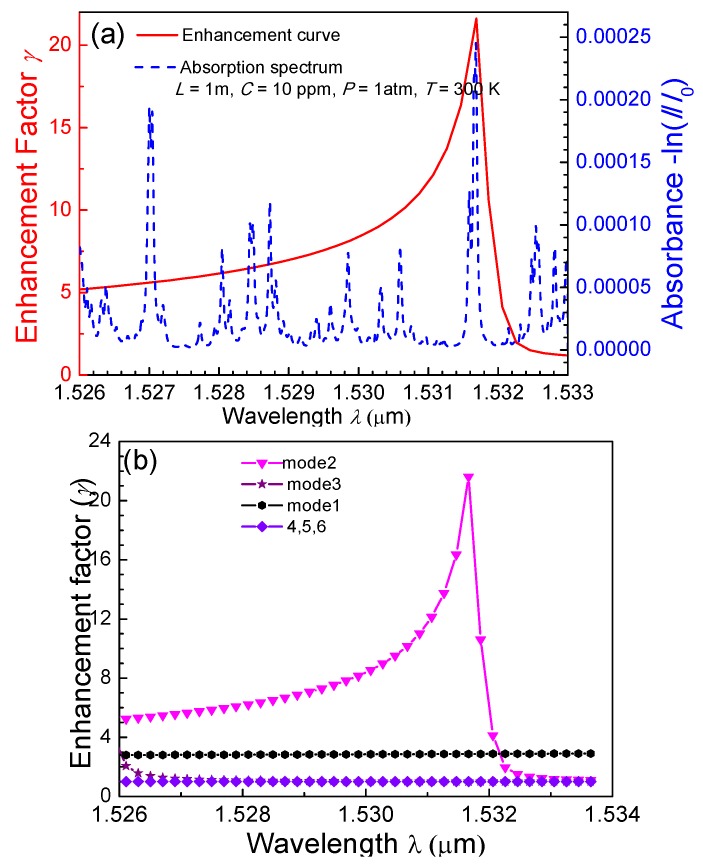
(**a**) Absorption spectrum of 1 ppm NH_3_ with a 1 m absorption length; curve of the enhancement factor *γ* for mode 3 versus the operating wavelength *λ*. (**b**) The relationship between the absorption wavelength of NH_3_ and the enhancement factor for modes 1−6.

NH_3_ was used as an example to explore the effects of slow light on gas detection. An absorption line located at 1.53168 µm was selected as the target line, with an absorption coefficient of 2.45332 × 10^−4^ m^−1^ and an extinction coefficient of 2.925 × 10^−5^. The mode coupling coefficients between the SMF mode and HC-PBF modes 1−6 were calculated to be 0.31, 0.32, 0.33, 0.71, 0.42, and 0.44, respectively, by using COMSOL software. To target the selected NH_3_ line and obtain the slow-light effect for mode 2 at 1.53168 µm, the refractive index of the liquid was determined to be 1.095. With these parameters, the absorption enhancement factor, as well as the absorption spectra of NH_3_, is shown in Figure 7a. It was found that the peak of the enhancement curve strictly corresponds to the absorption line at 1.53168 µm. The relationship between the enhancement factor and absorption wavelength is shown in Figure 7b. Only modes 1–3, especially mode 2, have an enhanced absorption effect.

**Figure 8:**
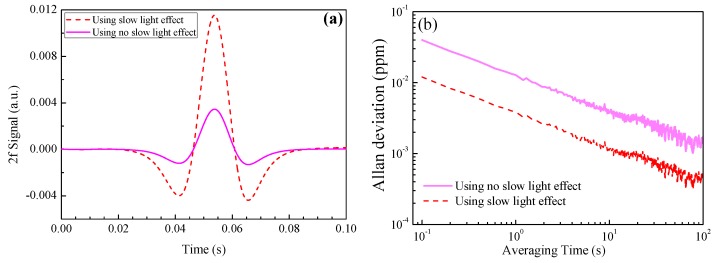
(**a**) The extracted 2f signals, both using the slow-light effect and without using such effect. (**b**) Allan deviation plots using the slow-light effect and without using such an effect.

As a comparison, under the cases of using the slow-light effect and without using such an effect, the extracted second harmonic (2*f*) signals are shown in Figure 8a, where a 1 ppm NH_3_ with a 1 m absorption length was used in the calculation. The 2*f* signal amplitude is enhanced by three times via the use of such an effect. When a white Gaussian noise (with a signal-to-noise ratio of 30 dB) was added in the sensor system, the Allan deviation plots for the two cases were as shown in Figure 8b. The Allan deviation was found to decrease from 0.04 ppm to 0.012 ppm by slow light-induced absorption enhancement, indicating a sensitivity enhancement by such an effect. Though PBF mode 2 has an enhancement factor of up to 20, the total enhancement factor is only 3, due to the inefficient coupling between the SMF mode and PBF mode 2, as well as the small gas absorption of other PBF modes.

## 5. Conclusions

A generic modeling and analysis of a slow-light enhanced absorption spectroscopic gas sensor using a mode-tuned HC-PBF was reported. By tuning the HC-PBF mode dispersion curve, a slow-light effect was achieved. The slow-light region can be tuned to an absorption line of a target gas species for absorption enhancement. A general rule of microfluidic parameters for targeting different gas species was obtained. The change in the 2*f* signal and the sensitivity enhancement resulting from the slow light effect were also investigated for NH_3_ as a test trace gas. The reported theoretical results will be useful in the development of other HC-PBF-based gas sensors.
